# Quality-of-life assessment may support the correct diagnosis of adult wheat allergy 

**DOI:** 10.5414/ALX02610E

**Published:** 2026-03-06

**Authors:** Florian Schusta, Anna Neyer, Sabine Dölle-Bierke, Josefine Grünhagen, Veronika Höfer, Margitta Worm

**Affiliations:** Division of Allergy and Immunology, Department of Dermatology, Venereology and Allergology, Charité – Universitätsmedizin Berlin, Corporate Member of Freie Universität Berlin and Humboldt-Universität zu Berlin, Berlin, Germany

**Keywords:** wheat allergy, food allergy, OFC, exercise, quality of life, cofactors

## Abstract

Background: Wheat is a frequent cause of food-induced allergic reactions in adults. In this study we aimed to assess the diagnostic value of skin prick test (SPT), specific immunoglobulin E (sIgE), but also quality of life in wheat-sensitized and allergic patients. Materials and methods: In this prospective, clinical study 80 patients were screened for eligibility. Subsequently, 36 wheat-sensitized patients underwent oral food challenges (OFCs) with supraphysiological amounts of gluten at rest and in combination with exercise. The challenge was stopped when objective symptoms occurred. Prior to the challenge, sIgE measurement and a SPT were conducted. The Food Allergy Quality of Life Questionnaire (FAQLQ), Food Allergy Independent Measure (FAIM), and Beck Anxiety Inventory (BAI) were used to assess the quality of life, perceived disease severity, and anxiety of the patients. Results: The OFC was performed with increasing amounts of gluten reaching a supraphysiological level. 24 patients (67%) were OFC positive with 21 reacting at rest. 3 patients reacted after the implementation of exercise. 60% of patients with a self-reported exercise dependency reacted in the OFC at rest. 8 of 21 patients who reacted at rest were rechallenged with exercise and lower doses of gluten, of which 5 reacted again. Exercise lowered the reaction threshold by 50% in these 5 patients. OFC-positive patients had stronger sensitization to gluten and its constituents and showed higher impairment in their quality of life and perceived burden of disease than OFC-negative patients. The receiver operator characteristics model including gluten SPT, omega-5-gliadin sIgE, and the FAIM score to predict OFC positivity yielded a 95.2% sensitivity and 83.3% specificity. Males displayed a higher degree of sensitization, but females had higher FAQLQ and FAIM scores. Conclusion: Although a high rate of exercise dependency was reported, most reactions were elicited at rest when the amount of gluten was upscaled. However, the eliciting amount and reaction threshold was lowered in the presence of exercise. Food allergy-related quality-of-life data can indicate psychological impairment due to the disease but may also serve as a patient-reported outcome tool which can support the diagnostic accuracy of wheat allergy.

## Introduction 

Although the prevalence of adult wheat allergy (aWA) in Europe is low, wheat flour is the most prevalent trigger of food-induced anaphylactic reactions in European adults [1, 2]. Wheat is comprised of non-gluten and gluten proteins. The former are subdivided into globulins and albumins while gluten comprises two distinct protein fractions: glutenins and gliadins [3]. Gliadins include omega-5-gliadin, the primary allergen associated with aWA [3, 4, 5]. Other allergens include α-/β-/γ-gliadins, high- and low-molecular-weight glutenins, and lipid-transfer-protein (LTP) [3, 6, 7, 8]. Recent studies have examined the significance of α-amylase inhibitors and hydrolyzed wheat proteins as allergens in aWA [8, 9, 10, 11]. Urticaria and cardiovascular symptoms are more common in aWA than in other food allergies. Compared with other food allergens, wheat is the most likely to cause severe anaphylaxis in adults [12]. The onset of symptoms in aWA patients is frequently prolonged, exceeding 30 minutes following ingestion [12, 13]. High doses that exceed the median daily gluten intake of an adult (median: 9.7 g) are often required to elicit a reaction [1, 12, 14]. The role of cofactors to elicit a reaction is high. Exercise has been reported as a predominant cofactor in aWA (≥ 80%) followed by alcohol and non-steroidal anti-inflammatory drugs (NSAID) [12, 13]. Cofactors can increase the severity of allergic reactions but also reduce the amount of allergen required to elicit a reaction [15, 16]. Considering this, the terms “Omega-5 gliadin allergy”, “wheat-dependent exercise-induced anaphylaxis”, and “wheat allergy dependent on augmentation factors” were implemented and are subject of recent deliberations regarding the appropriate nomenclature [13, 17, 18]. aWA patients exhibit a diminished health-related quality of life (QoL) that has been shown to improve after confirmation of the diagnosis [19, 20, 21]. Besides the adult form of the Food Allergy Quality of Life Questionnaire (FAQLQ-AF), a standardized, patient-related assessment tool in food allergy, the perceived disease severity of food-allergic patients can be determined by the adult form of the Food Allergy Independent Measure (FAIM-AF). aWA patients have been shown to exhibit high FAIM scores indicating a high perceived disease severity [19]. Compared with other food allergies, aWA has been shown to have the greatest negative social impact and results in a most restrictive diet for those affected [22, 23]. Subsequently, aWA patients present with higher FAIM scores compared with other food-allergy patients [22, 23]. The diagnosis of aWA should be made according to the guidelines [24]. A detailed allergological history is followed by a skin prick test (SPT) with wheat extract, native wheat flour, or native wheat gluten [24, 25]. Furthermore, specific immunoglobulin E (sIgE) to wheat, gluten, omega-5-gliadin, α-/β-/γ-gliadin, and wheat LTP should be measured in the serum as well as basal tryptase levels [5, 6, 8, 9]. The final diagnostic pillar is the oral food challenge (OFC) performed with gluten in titrated steps in a clinical setting [16, 24, 25]. The objective of this study was to evaluate the relevance of exercise as the primary cofactor in aWA. Second, to assess sensitization profiles and the QoL of patients undergoing OFC. Last, the aim was to identify predictors of clinically relevant aWA. 

## Materials and methods 

To assess the clinical reaction patterns of wheat-sensitized adults with confirmed or suspected aWA, we performed OFCs with gluten in combination with exercise in the outpatient clinic between December 2021 and April 2024. This prospective study was part of a multicenter wheat allergy study project (Wheat-a-Baic). This study was approved by the ethics committee of Charité – Universitätsmedizin Berlin (EA2/203/21) and is registered in the German Clinical Trials Register (DRKS00027174). 

### Eligibility and exclusion criteria 

Patients aged 18 – 68 years with a positive history (e.g., reproducible symptoms after the consumption of wheat-containing products or a history of anaphylaxis to wheat) and a sensitization to wheat (SPT to wheat flour/gluten ≥ 3 mm or sIgE ≥ 0.35 kU/L to wheat flour, gluten, α-/β-/γ-gliadins, omega-5-gliadin, or lipid-transfer-protein) were eligible for participation and gave written informed consent. Patients with a severe systemic disease or an acute infection were excluded. Furthermore, the use of certain medications (e.g., betablockers), pregnancy, or breastfeeding led to exclusion. 

### SPT and in-vitro diagnostics 

A standardized diagnostic protocol was implemented for all recruited patients. Wheat flour and purified gluten flour (Kröner Stärke, Ibbenbüren, Germany) were mixed 1 : 5 with saline solution and used for SPT. Skin reactions ≥ 3 mm were considered positive. sIgE against wheat flour (f4), gluten (f79), α-/β-/γ-gliadins (f416), omega-5-gliadin (rTri a 19), wheat LTP (rTri a 14), total IgE, and tryptase were measured using the ImmunoCAP system (ThermoFisher, Uppsala, Sweden). 

### OFC 

The OFC followed a modified version of the established OFC protocol by Christensen et al. [15] and was unblinded. Gluten buns were prepared by mixing purified gluten flour (Kröner Stärke) and twice the amount of water. The resulting dough was then formed into buns and baked for 15 minutes at 200 °C. A cumulative amount of 56 g of gluten (8 – 16 – 32 g) was orally administered on 1 day at rest in 90-minute intervals. If no reaction occurred, the same amount of gluten was prepared for the OFC with exercise. If the patient reacted in the OFC at rest, less gluten (2 + 4 g + exercise or 8 + 12 g + exercise) was administered on day 2. A physical examination including vital parameters and a lung function test preceded the tests. Exercise was implemented by bike ergometry at increasing wattage and after each gluten dose. Maximal wattage levels were calculated for each patient based on their self-declared fitness level, age, sex, and weight. The provocation was stopped when objective anaphylactic symptoms occurred [26]. Symptoms were classified using the modified Sampson score [27], the Muraro score [28], and the numeric Food Allergy Severity Score (nFASS) [29]. 

### Questionnaires 

The questionnaires used included the German versions of the FAQLQ-AF [30, 31], the FAIM [30, 32], and the Beck Anxiety Inventory (BAI) [33]. The FAQLQ-AF covers 29 items used to calculate a total score, while selected items can be grouped to determine four domain scores: allergen avoidance and dietary restrictions (AADR), emotional impact (EI), risk of accidental exposure (RAE), and food allergy-related health (FAH). The FAIM-AF was developed to assess the construct validity of the FAQLQ-AF. Six items measure the expectation of outcome after ingesting the allergen as well as allergy-related dietary and social restrictions (“How big do you think the chance is that you… (1) will accidentally eat something to which you are allergic?”, (2) “… will have a severe reaction if you accidentally eat something to which you are allergic?”, (3) “… will die if you accidentally eat something to which you are allergic?”, (4) “… can not do the right things for your allergic reaction, should you accidentally eat something to which you are allergic?”, (5) “How many foods are you unable to eat because of your food allergy?”, (6) “How much does your food allergy affect things you do with others?”) [32]. The final scores both range from 1 to 7 with higher scores indicating greater impairment of QoL (FAQLQ-AF, domain scores) or high perceived disease severity (FAIM) [31, 32]. To determine anxiety, the BAI utilizes 21 items which can be scored between 0 and 3. Based on this scoring, anxiety levels can be differentiated as minimal (0 – 7), mild (8 – 15), moderate (16 – 25), and severe anxiety (26 – 63) [33]. 

### Statistics 

IBM SPSS Statistics for Windows was utilized to conduct the statistical analysis (IBM SPSS Statistics, Version: 29.0.0.0 (241), IBM, Armonk, NY, USA). We performed a descriptive analysis and used the Mann-Whitney U test with a confidence level of 95% (p < 0.05) to determine statistical significance. The confidence interval was set at 95% (p ≤ 0.05). Receiver operator characteristics (ROC) curves were used to obtain cut-off values, sensitivity, and specificity. 

## Results 

**Figure 1.**
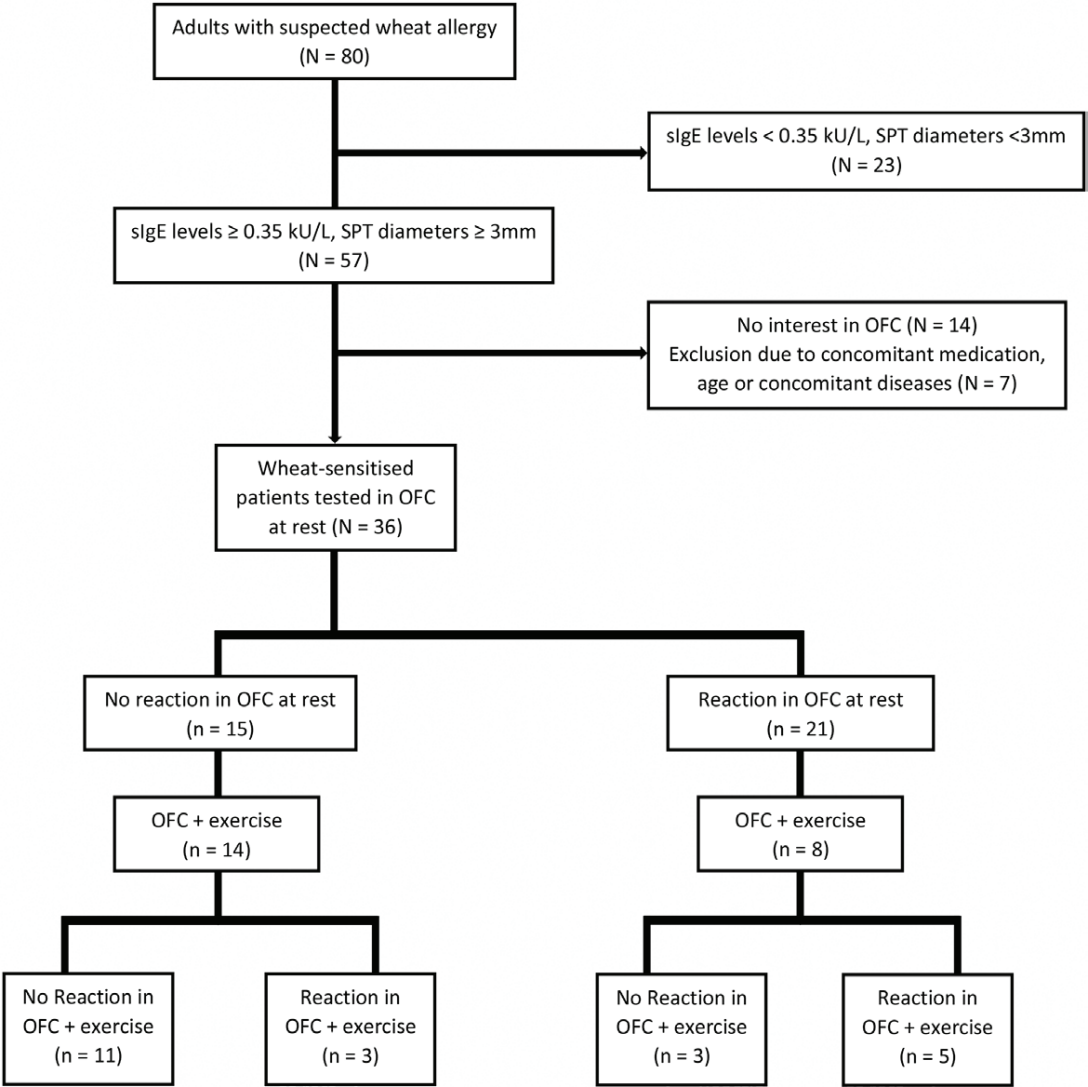
Flowchart of patient recruitment and OFC results. (OFC = oral food challenge; SPT = skin prick test; sIgE = specific Immunoglobulin E).

80 individuals were screened for eligibility. Of these, 36 wheat-sensitized individuals were challenged. The OFCs at rest (n = 36) and the re-OFCs with exercise of initially OFC-negative patients (n = 14) led to the identification of 24 OFC-positive aWA patients (67%) in our cohort (Figure 1). Both groups of OFC-positive and -negative patients show an equal gender distribution and were comparable in mean age. Further demographics and clinical characteristics are listed in Table 1. 

### Reported vs. observed cofactor dependency and OFC results 

Most reactions (n = 21; 87.5%) in the OFC occurred at rest with supraphysiological doses. Only 3 patients (12.5%) needed these high doses (56 g) plus exercise to react. A more detailed overview of the OFC results is presented in Figure 2a. Prior to the OFCs, 21/35 patients (60%) reported a dependency on a cofactor to elicit a reaction. The most frequently suspected was exercise (20/21; 95.2%), followed by stress (12/21; 57.1%) and alcohol (3/21; 14.3%). Two patients each (9.5%) identified NSAIDs, sleep deprivation, and menstruation as cofactors, while 1 patient each (4.8%) noted an influence of temperature change and concomitant infection. Real-life, systemic, allergic reactions to wheat were more prevalent in cofactor-dependent patients (19/21; 90.5%) than in cofactor-independent patients (9/14; 64,3%). Most patients who reported an exercise dependency had a reaction to gluten even in the absence of exercise (12/20; 60%). This rate is comparable to that observed in cofactor-independent patients (8/15; 53.3%). The mean amount of gluten required to elicit a reaction in exercise-dependent patients was higher (22.67 g; n = 12) than in cofactor-independent patients (16 g; n = 8). The 7 cofactor-independent patients who tolerated 56 g of gluten at rest retained this tolerance to the same amount when the OFC was performed with exercise. Eight patients who were positive in an OFC at rest were retested with a reduced amount of gluten and exercise. Three patients subsequently tested negative while 5 patients reacted positively again in the OFC to a lower amount. The mean reaction-eliciting amount of gluten was decreased by 50% (from 11.2 to 5.6 g) in these patients. In 2 patients, exercise was newly discovered as a cofactor (Figure 2b). 

### SPT and in-vitro diagnostics 

Patients with a positive OFC were stronger sensitized than OFC-negative patients (Table 2). SPT wheal sizes (mm) to wheat (6.0 vs. 3.0, p = 0.0063) and gluten (5.5 vs. 2.75, p = 0.0009), the sIgE levels (kU/L) to gluten (4.72 vs. 0.27, p = 0.0004), α-/β-/γ-gliadins (2.89 vs. 0.10, p = 0.0002), and omega-5-gliadin (6.22 vs. 0.10, p=0.0013) were higher in the OFC-positive than the OFC-negative group. However, the profiles of wheat sensitization differed between the groups: Most OFC-positive patients exhibited sensitization to gluten in both the SPT and sIgE test (20/22, 90.9%), followed by wheat in the SPT (19/22, 86.4%) and to omega-5-gliadin sIgE (20/24, 83.3%). By contrast, the strongest sensitization among OFC-negative patients was observed for wheat sIgE (9/10, 90%) followed by wheat and gluten SPT (6/12, 50%). A minority of OFC-negative patients showed positive sIgE against gluten (5/12, 41.7%), α-/β-/γ-gliadins (1/12, 8.3%), and omega-5-gliadin (2/12, 16.7%).The median sIgE levels to LTP were low (both 0.10 kU/L) in both groups, and only 3 patients overall were sensitized (OFC-positive: 1/23, 4.3% / OFC-negative: 2/12, 16.7%). 

### QoL and anxiety 

Patients with a positive OFC are more severely impaired regarding their QoL than those with a negative OFC (median FAQLQ-AF score: 4.66 vs. 3.30). This was also observed across all domain scores with median values of 4.5 – 5.0 in the OFC positive group and scores of 2.33 – 3.87 in the OFC negative group (Table 2). The median domain scores of EI (5.00 vs. 3.5) and RAE (4.75 vs. 2.33) show the largest difference between OFC-positive and OFC-negative patients. However, only the FAIM score demonstrated statistical differences between the groups, indicating a higher perceived disease severity in OFC-positive patients (4.33 vs. 3.25, p = 0.0033). Median anxiety, measured by the BAI, was mild (11) in OFC-positive patients and negligible (5) in OFC-negative patients. 

### Impact of sex in wheat-allergic and wheat-sensitized individuals 

Male OFC-positive patients had a higher degree of sensitization in all wheat-specific IgE results (except LTP), but also higher total IgE compared to female OFC-positive patients (total IgE (kU/L): 406.50 vs. 186.50). Significant differences are seen for both α-/β-/γ-gliadins sIgE (p = 0.0210) and omega-5-gliadin sIgE (p = 0.0046). Additionally, the median SPT wheal size to wheat (7.5 vs. 5.0 mm) and gluten (7.5 vs. 4.5 mm, p = 0.0132) was increased in OFC-positive males. Finally, OFC-positive women experienced a lower QoL than OFC-positive men (median FAQLQ-AF score, 5.46 vs. 3.88, p = 0.0013). Significantly higher scores in OFC-positive women were also observed in the domains AADR (p = 0.0047), EI (p = 0.0323), and RAE (p = 0.0008). Female OFC-positive patients also present a higher perceived disease severity, indicated by a significantly higher FAIM score (4.58 vs. 3.67, p = 0.0037). The anxiety levels of both sexes were comparable (Supplemental Table S1). 

### Predictability of positive OFC outcome 

ROC curves were constructed to determine the AUC and optimal cut-off values of selected variables in relation to OFC positivity (Figure 3). The sIgE against α-/β-/γ-gliadins exhibited a high AUC (88.8%) and the best specificity (91.7%) at a cut-off value of 0.24 kU/L. A high sensitivity (90.9%) was demonstrated by gluten sIgE at a cut-off value of 0.69 kU/L, corresponding to an AUC of 87.7%. The optimal combination of variables was identified as gluten SPT, omega-5-gliadin sIgE, and the FAIM score. This resulted in the highest sensitivity (95.2%), a high specificity (83.3%), and the highest AUC (94.8%). The addition of the FAIM score to “Gluten SPT + O5G” enhanced sensitivity by 13.4% and expanded the AUC by 8.4% (Supplemental Table S2). 

## Discussion 

In this study we prospectively identified a cohort of adult aWA patients. Sensitized individuals underwent OFCs and were analyzed regarding clinical characteristics, OFC reaction profile, and QoL. Gender distribution among our cohort was equal. Most patients reported a cofactor dependency to elicit reactions, with exercise being the most common. This underlines the high prevalence of cofactors in aWA in general and of exercise in particular [12, 13]. The second most prevalent reported cofactor in our cohort was stress followed by alcohol. While alcohol is commonly named as a cofactor, stress did not display the same prevalence in other cohorts [13, 20]. 

The mean reaction-eliciting dose of gluten in the OFCs at rest was 20.2 g supporting previous data that supraphysiological amounts of gluten exceeding the median daily gluten intake of 9.7 g are needed to elicit a reaction in aWA patients [1, 12, 14]. This high amount of pure gluten resulted in reactions at rest in 60% of patients who had previously only experienced exercise-induced reactions. Only 3 patients in our cohort exhibited exclusively exercise-induced reactions, and this occurred only after ingestion of 56 g of gluten and 3 exercise sessions. This reinforces the necessity of deliberations regarding the appropriate nomenclature for aWA, as exercise is not required to cause a reaction when gluten doses are sufficiently high [15, 17, 18, 25]. Although most consumers’ daily gluten intake does not yet exceed 10 g, the demand for plant-based food alternatives has led to food items with a high gluten content (e.g., seitan, meat substitutes), becoming more readily available, which may pose a risk to aWa patients [14]. In 25 of 29 positive OFCs (86.2%), the latency period after the last dose and the onset of symptoms exceeded 30 minutes thus confirming another characteristic of aWA [12, 13]. 

The rate of self-reported systemic allergic reactions prior to the OFC was higher in cofactor-dependent aWA patients than in those not dependent on cofactors. Of the 14 patients who declared a non-cofactor dependency prior to the OFC, we nevertheless identified 7 (50%) with a non-cofactor-dependent aWA. A difference in severity was not confirmed in the OFCs (Figure 2a). While the severity of the reaction remained unchanged, the amount of gluten required to elicit a reaction declined by 50% in 5 patients when exercise was introduced (Figure 2b). Nevertheless, not all patients are aware of cofactors. In 2 patients, exercise was an unrecognized cofactor. These findings are consistent with previous studies which suggest that cofactors reduce the amount of gluten required to elicit a reaction and thus help to identify new and relevant cofactors [15, 16, 25]. 

One patient exhibiting a severe reaction to 24 g of gluten at rest did not develop symptoms in an OFC involving 20 g of gluten combined with exercise even though “exercise” was suspected as a cofactor by the patient prior to the OFC. This illustrates the potential influence of additional, yet unidentified cofactors (e.g., stress, hormonal levels) on intraindividual reaction thresholds. Such factors may contribute to the significant discrepancy between the high gluten amounts required to elicit a reaction in a controlled clinical setting and the much smaller gluten doses capable of eliciting reactions in uncontrolled real-life situations [25]. 

The sIgE sensitization profile of OFC-positive patients in our study confirms that omega-5-gliadin is a dominant, sensitizing allergen in aWA. Omega-5-gliadin and α-/β-/γ-gliadins sIgE was detected in 83.3%/77.3% of OFC-positive patients and only in 16.7%/8.3% of OFC-negative patients, with median sIgE levels of 6.22/2.89 kU/L and 0.10/0.10 kU/L. This underlines the role of gliadins, especially omega-5-gliadin as the major allergen in aWA [3, 4, 5]. When further differentiated by sex, it is evident that female OFC-positive patients present with lower rates of sensitization in SPT and sIgE and lower specific and total IgE levels overall. However, the rate of positive OFC was equal between the sexes. This finding is in line with previous data that female sex hormones may promote allergic reactions. This may increase the risk of food allergies in women, despite their reduced sensitization compared to men [34]. 

Suffering from food allergy, and its subsequent day-to-day management, can impact the lifestyle. It has the potential to negatively affect the QoL of patients [21]. OFC-positive aWA patients, who report a higher rate of systemic reactions in the past, present with higher total FAQLQ scores than OFC-negative patients (total FAQLQ: 4.66 vs. 3.30). It is known that systemic reactions can reduce QoL, but having the diagnosis for more years can, on the other hand, improve it [21]. A smaller yet similar difference of the total FAQLQ was reported for a food-allergic, OFC-tested cohort (4.56 (OFC-positive) vs. 4.24 (OFC-negative)) [35]. The total FAQLQ and the domain scores of the OFC-positive group (4.66) are comparable to those of one previously reported cohort (4.70) and higher than in two other cohorts examining aWA (4.20 and 3.65) [19, 20, 36]. Studies with nut-allergic patients revealed higher QoL [37], but also comparable numbers with this cohort [36]. Patients with birch-pollen associated food allergy were reported to have better QoL scores than aWA patients [30]. Our finding that female aWA patients experience a significantly reduced QoL compared with men (median FAQLQ-AF score: 5.46 vs. 3.88, p = 0.0013) is in accordance with Zubrinich et al. [36] who reported a mean total FAQLQ score of 4.75 in female and 4.06 in male patients in a cohort of individuals with wheat and peanut allergy. Like our cohort, the EI and RAE domain scores were significantly higher in women [36]. 

We identified higher FAIM scores in OFC-positive versus OFC-negative patients. This supports data from other studies reporting high FAIM scores in aWA patients, with scores exceeding those of patients with other food allergies [19, 22, 23, 35, 37]. One explanation might be that aWA patients display heterogeneous disease profiles and repeatedly suffer from real-life reactions, possibly due to changing reaction thresholds and unrecognized cofactors. In addition, aWA patients often experience prolonged diagnostic workup times of their disease and may receive no or inadequate dietary advice, leading to dietary and social restrictions [22, 23, 36]. The addition of the FAIM score to the standard diagnostic variables “gluten SPT” and “omega-5-gliadin sIgE” in the ROC analysis for a positive OFC increased the AUC by 8.4 to 94.8% and the sensitivity by 13.4 to 95.2% at cut-off values of 3.75 mm (gluten SPT), 0.98 kU/L (omega-5-gliadin sIgE) and 3.58 (FAIM score) while specificity remained unchanged. Based on these findings, the FAIM score may serve as a simple, quick, and non-invasive addition to standard diagnostic procedures such as SPT and sIgE testing, helping to support the often challenging diagnostic process in aWA. 

Our study has several limitations. First, it is a single-center study with a relatively small cohort. Although this is often the case in aWA studies due to the low prevalence of diagnosed aWA patients, more multi-center studies are warranted. Second, we did not perform double-blind, placebo-controlled food challenges. The unique texture of gluten bread makes a placebo control difficult to implement. Third, the results of the ROC models used in this study must be considered exploratory as they were performed with a small number of patients. Fourth, the use of supraphysiological doses of gluten in an OFC does not represent daily amounts of gluten necessary in real life. Unidentified and inter-individual cofactors that lower the gluten threshold in real life, which cannot be reproduced in an OFC setting, may therefore lead to false-negative OFC results. Last, the implementation of the FAIM score as a supporting diagnostic tool needs to be further evaluated. Patients who only experience mild or subjective wheat-dependent symptoms but do not fulfil the criteria of a positive OFC may also present with a high perceived disease severity while not allergic to wheat. Hence, SPT and in-vitro diagnostics are indispensable. Moreover, the FAIM score is not an aWA-specific questionnaire. Nevertheless, the FAIM scores of aWA patients are higher in comparison to other food allergies [22, 23]. Therefore, the FAIM score has the potential to serve as an additional diagnostic tool for clinicians to identify patients with suspected aWA, but further evaluation in future studies is necessary. 

In conclusion, OFC reactions in aWA patients can be induced at rest with supraphysiological amounts of gluten and independently of the patient’s cofactor dependence. The implementation of exercise in the OFCs lowered the reaction threshold in a subgroup of aWA patients by 50%. OFC-positive patients were stronger sensitized to gluten and its constituents, had an impaired QoL, and a higher perceived disease severity and anxiety level than OFC-negative patients. This underscores the significance of professionals providing comprehensive patient education in identifying foods high in gluten (e.g., seitan) and in mitigating the effects of potential cofactors in everyday life. Consequently, patients are better informed about their condition. This can promote an increase of QoL and a reduction in perceived disease severity and anxiety. The assessment of QoL should be regularly implemented in the diagnostic workup of aWA as it can contribute to a higher accuracy of SPT and sIgE and most importantly can support recognition of psychological distress in these patients. Further studies should evaluate an allergen-specific QoL instrument to support clinicians and better respond to patients’ needs. 

## Acknowledgments 

We thank all our study participants for taking part in the study. We thank G. Bindke and A. Alexiou for the clinical supervision of the participants, A. Pöhlmann for the statistical support, and K. Weigt for the technical support. Open Access funding is enabled and organized by Projekt DEAL. 

## Authors‘ contributions 

F.S. contributed to the development of the provocation scheme, organized patient recruitment, performed the clinical study as well as the statistical analyses, designed the figures, and wrote the manuscript. A.N. contributed to patient recruitment, performed the clinical study and reviewed and edited the manuscript. S.D.B. contributed to the development of the study design and the provocation scheme and reviewed and edited the manuscript. J.G. contributed to the development of the provocation scheme, supported the clinical study, and reviewed and edited the manuscript. V.H. supported the clinical study and reviewed and edited the manuscript. M.W. supervised the study, contributed to the development of the study design as well as the provocation scheme, supervised the analyses of the results, reviewed the initial draft, and assisted in writing the manuscript. 

## Funding 

This work was supported by German Federal Ministry of Education and Research (BMBF-Wheat-a-Baic FKZ- 01EA2001A). 

## Conflict of interest 

All authors state no conflicts of interest. 

**Table 1.** Demographics and clinical characteristics of OFC-positive and OFC-negative patients.

	**Positive (n = 24; 67%)**	**Negative (n = 12; 33%)**
Age (mean, range)	40 years (± 1 2), 19 – 61	37 years (± 15), 20 – 61
Sex	Female: n = 12 (50%) Male: n = 12 (50%)	Female: n = 6 (50%) Male: n = 6 (50%)
Wheat allergy status	Suspected: n = 2 (8%) Physician confirmed: n = 22 (92%)	Suspected: n = 4 (33%) Physician confirmed: n = 8 (67%)
Self-reported systemic reaction to wheat	No: n =2 (8%) Yes: n = 22 (92%)	No: n = 5 (42%) Yes: n = 7 (58%)
Cofactor-dependent wheat allergy	No: n = 7 (30%) Yes: n = 16 (70%)	No: n = 7 (58%) Yes: n = 5 (42%)
Sampson-Score pre OFC (1 – 5) Muraro-Score pre OFC (1 – 3)	Median: 4 (range: 1 – 4) Median: 3 (range: 1 – 3)	Median: 3.5 (range: 3 – 4) Median: 3 (range: 3 – 3)
Life years with diagnosis	Median: 2.49 years Range: 0.05 – 11.03 years	Median: 4.05 years Range: 0.19 – 20.76 years
Atopic comorbidities	Allergic rhinoconjunctivitis (n = 6), asthma (n = 1), eczema (n = 3)	Allergic rhinoconjunctivitis (n = 8), asthma (n = 0), eczema (n = 0)
Elicitors of allergic rhinoconjunctivitis	Grass pollen (n = 6), weed pollen (n = 5), animals (n = 1), house dust mite (n = 1), mold (n = 1)	Grass pollen (n = 7), weed pollen (n =7), animals (n = 5), house dust mite (n = 4), mold (n = 2)
Avoidance of other cereals and cultivars than common wheat	Spelt (n = 9), rye (n = 7), emmer (n = 3), einkorn (n = 1), oat (n = 2), barley (n = 2)	All cereal (n = 3), spelt (n = 2), rye (n = 2)

OFC = oral food challenge.

**Figure 2a.**
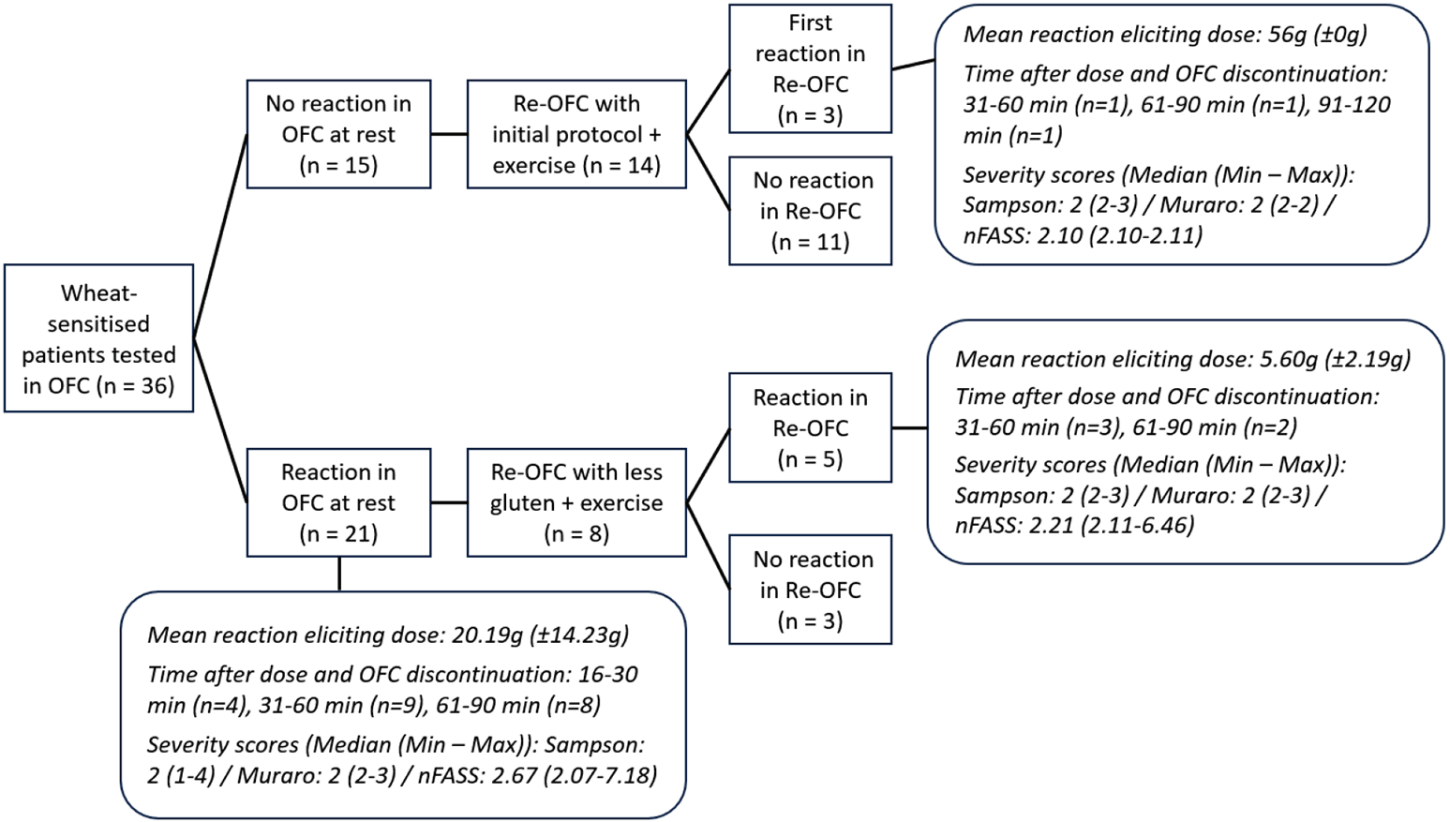
Flowchart of OFC results (nFASS = numeric Food Allergy Severity Score; OFC = oral food challenge).

**Figure 2b.**
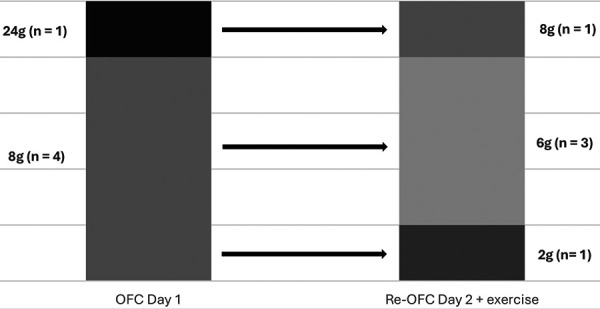
Reaction-eliciting amounts of gluten (in g) of 5 patients who reacted both at rest and in combination with exercise. (OFC = oral food challenge).

**Table 2.** Median skin prick test and IgE values and median FAQLQ-AF, FAIM, and BAI scores of OFC-positive and OFC-negative patients.

	**Median values (IQR)**		**p-value**
	**OFC positive ** **(n = 24; 67%)**	**OFC negative ** **(n = 12; 33%)**	
Skin prick test (in mm), positive: ≥ 3 mm			
Wheat	6.0 (3.63) Positive 19/22 (86.4%)	3.0 (5.75) Positive 6/12 (50%)	**0.0063**
Gluten	5.5 (3.63) Positive 20/22 (90.9%)	2.75 (3.88) Positive 6/12 (50%)	**0.0009**
Histamine	7.25 (2.63)	7.25 (2.75)	0.7166
IgE measurement (in kU/L) and tryptase (in µg/L), positive: ≥ 0.35 kU/L			
Total IgE	272 (567.50)	354 (413.00)	1.0000
Wheat (f4)	0.84 (3.23) Positive 17/23 (73.9%)	0.68 (2.24) Positive 9/10 (90%)	0.8400
Gluten (f79)	4.72 (12.48) Positive 20/22 (90.9%)	0.27 (0.96) Positive 5/12 (41.7%)	**0.0004**
α-. β-. γ-gliadin (f98)	2.89 (8.55) Positive 17/22 (77.3%)	0.10 (0.09) Positive 1/12 (8.3%)	**0.0002**
Omega-5-gliadin (rTria 19. f416)	6.22 (13.79) Positive 20/24 (83.3%)	0.10 (0.00) Positive 2/12 (16.7%)	**0.0013**
LTP (rTria 14)	0.10 (0.00) Positive 1/23 (4.3%)	0.10 (0.05) Positive 2/12 (16.7%)	0.2090
Tryptase preOFC	4.62 (2.17)	4.12 (2.37)	0.0953
FAQLQ scores			
Total FAQLQ	4.66 (1.80)	3.30 (2.78)	0.0700
Allergen avoidance and dietary restrictions	4.50 (2.23)	3.87 (2.76)	0.2334
Emotional impact	5.00 (2.25)	3.50 (3.14)	0.0533
Risk of accidental exposure	4.75 (1.72)	2.33 (4.44)	0.0866
Food-allergy related health	4.50 (2.83)	3.33 (2.08)	0.3846
FAIM score			
FAIM	4.33 (1.17)	3.25 (1.42)	**0.0033**
BAI			
BAI-Score	11 (20)	5 (15)	0.1775

IQR = interquartile range; OFC = oral food challenge; IgE = immunoglobulin E; FAQLQ = Food Allergy Quality of Life Questionnaire; FAIM = Food Allergy Independent Measure; BAI = Beck anxiety inventory. Bold = p ≤ 0.05..

**Figure 3.**
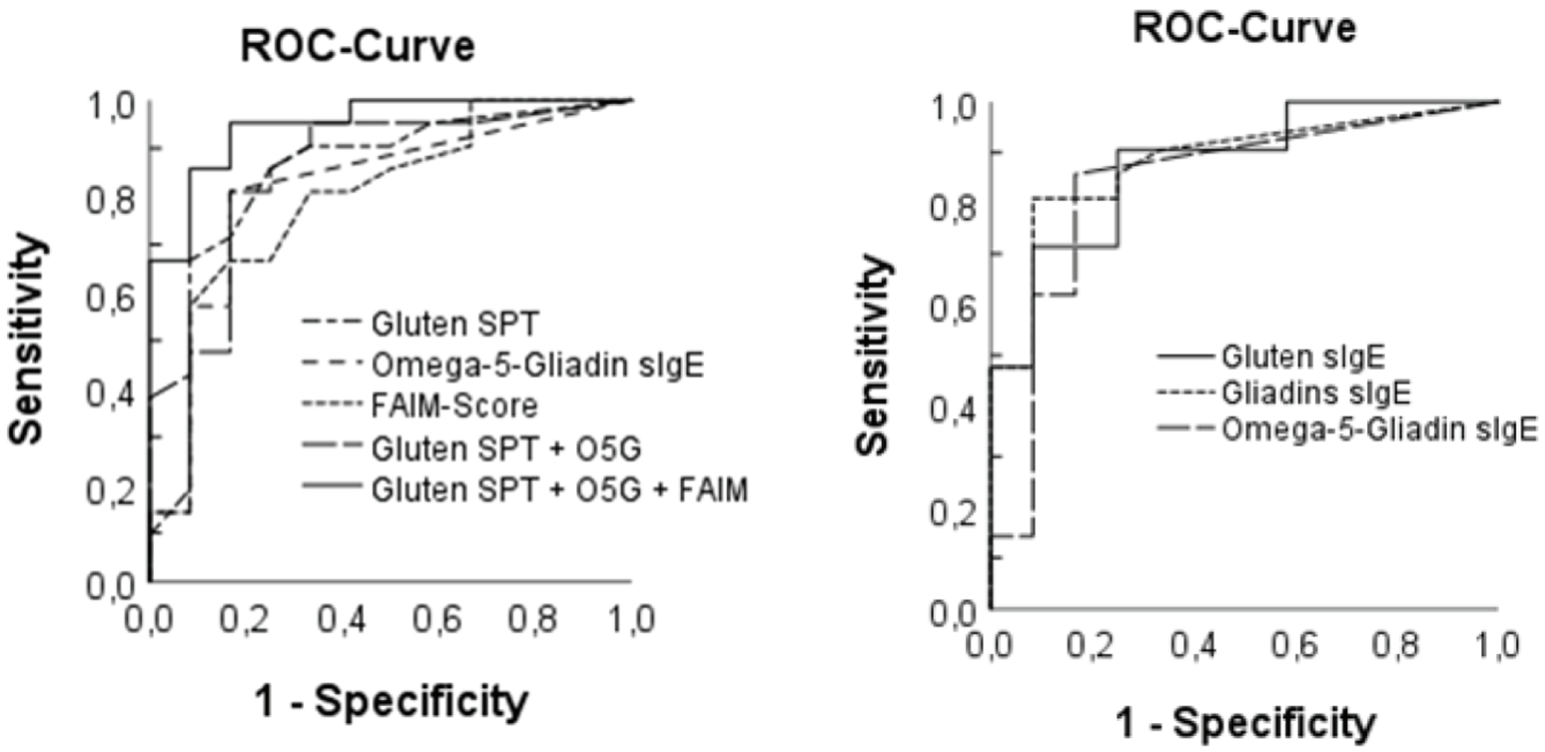
ROC curves of different variables in relation to OFC positivity (SPT = skin prick test; sIgE = specific Immunoglobulin E; O5G = omega-5-gliadin sIgE; FAIM = Food Allergy Independent Measure).

## Supplemental material

Supplemental material Supplemental Tables
