# Supplementary material for: Quality-of-life assessment may support the correct diagnosis of adult wheat allergy
Source: Allergol Select. 2026 Mar 6;10:16–27. doi: 10.5414/ALX02610E (PMC12980459; doi:10.5414/ALX02610E)
Supplement: Supplemental material [file allergologieselect-10-016-S01.pdf]

Supplemental material

Supplemental Table SI: Median SPT and IgE values and median FAQLQ-AF, domain, FAIM and BAI scores of OFC-positive female and male patients.

	Median values (IQR)		p-value
	Female/OFC + (n = 12; 50%)	Male/OFC + (n = 12; 50%)	
Skin-Prick-Test (in mm), positivity = ≥ 3mm			
Wheat	5.0 (5.0) Positive 8/12 (66.7%)	7.5 (3.5) Positive 11/12 (91.7%)	0.1382
Gluten	4.5 (3.5) Positive 9/12 (75%)	7.5 (3.0) Positive 11/12 (91.7%)	0.0132
Histamine	7.0 (3.5)	7.5 (2.0)	0.2745
IgE measurement (in kU/L) and tryptase (in µg/L), positivity = ≥ 0.35 kU/L			
Total IgE	186.5 (317.08)	406.5 (813.75)	0.0735
Wheat (f4)	0.44 (1.07) Positive 7/12 (58.3%)	2.25 (2.97) Positive 10/12 (83.3%)	0.1568
Gluten (f79)	1.35 (5.96) Positive 8/12 (66.7%)	9.25 (10.00) Positive 12/12 (100%)	0.0649
α-. β-. γ-gliadin (f98)	1.23 (3.10) Positive 6/12 (50%)	8.11 (7.55) Positive 11/12 (91.7%)	0.0210
Omega-5-gliadin (rTria 19. f416)	2.55 (6.10) Positive 8/12 (75%)	15.10 (10.21) Positive 12/12 (100%)	0.0046
LTP (rTria 14)	0.10 (0.00) Positive 1/12 (8.3%)	0.10 (0.00) Positive 0/12 (0%)	0.8998
Tryptase preOFC	4.72 (4.26)	4.21 (2.31)	0.6659
FAQLQ-scores			
Total FAQLQ	5.46 (0.80)	3.88 (1.40)	0.0013
Allergen avoidance and dietary restrictions	5.60 (1.68)	3.59 (1.59)	0.0047
Emotional impact	5.76 (1.75)	4.43 (1.68)	0.0323
Risk of accidental exposure	5.44 (1.06)	3.75 (1.94)	0.0008
Food-allergy related health	4.67 (1.92)	3.33 (3.33)	0.4656
FAIM-score			
FAIM	4.58 (1.00)	3.67 (0.83)	0.0037
BAI			
BAI-Score	10 (23)	12 (21)	0.7318

IQR = interquartile range; OFC = oral food challenge; IgE = immunoglobulin E; FAQLQ = Food Allergy Quality of Life Questionnaire; FAIM = Food Allergy Independent Measure; BAI = Beck anxiety inventory.

Supplemental Table S2. AUCs, Cut-off values, sensitivity and specificity of variables assessed with ROCs.

	n	AUC	Cut-off value	Sensitivity	Specificity	p-value
Gluten SPT	34	84.8%	3.75 mm	86.4%	75.0%	0.0009
Gluten sIgE	34	87.1%	0.69 kU/L	90.9%	75.0%	0.0004
α-. β-. γ-gliadins sIgE	34	88.8%	0.24 kU/L	81.8%	91.7%	0.0002
Omega-5-gliadin sIgE	36	82.3%	0.98 kU/L	83.3%	83.3%	0.0019
FAIM-score	35	80.6%	3.58	82.6%	66.7%	0.0033
Gluten SPT + Omega-5-G sIgE	34	86.4%	NA	81.8%	83.3%	0.0005
Gluten SPT + Omega-5-G sIgE + FAIM-score	33	94.8%	NA	95.2%	83.3%	0.0000

AUC = area under the curve; SPT = skin prick test; sIgE = specific Immunoglobulin E; FAIM = Food Allergy Independent Measure.

Schusta F, Neyer A, Dölle-Bierke S, Grünhagen J, Höfer V, Worm M.
Quality-of-life assessment may support the correct diagnosis of adult wheat allergy.
Allergol Select. 2026; *10:* 16-27.
DOI 10.5414/ALX02610E
citation
